# PD1 signal transduction pathways in T cells

**DOI:** 10.18632/oncotarget.17232

**Published:** 2017-04-19

**Authors:** Hugo Arasanz, Maria Gato-Cañas, Miren Zuazo, Maria Ibañez-Vea, Karine Breckpot, Grazyna Kochan, David Escors

**Affiliations:** ^1^ Immunomodulation Group, Navarrabiomed-Biomedical Research Centre, IdISNA, Pamplona, Spain; ^2^ Rayne Institute, Division of Infection and Immunity, University College London, London, United Kindom; ^3^ Laboratory of Molecular and Cellular Therapy Department of Biomedical Sciences, Vrije Universiteit Brussel, Brussels, Belgium

**Keywords:** PD-L1, PDL1, PD1, B7-H1, cancer

## Abstract

The use of immune checkpoint inhibitors for the treatment of cancer is revolutionizing oncology. Amongst these therapeutic agents, antibodies that block PD-L1/PD1 interactions between cancer cells and T cells are demonstrating high efficacies and low toxicities. Despite all the recent advances, very little is yet known on the molecular intracellular signaling pathways regulated by either PD-L1 or PD1. Here we review the current knowledge on PD1-dependent intracellular signaling pathways, and the consequences of disrupting PD1 signal transduction.

## INTRODUCTION

Anti-cancer immunotherapies are finally becoming clinically efficacious after many decades of intense research and development. Amongst these, antibody-mediated disruption of programmed death ligand 1 (PD-L1)/programmed death receptor 1 (PD1) interactions is one of the most efficacious with milder adverse effects than chemo- and radiotherapy [1]. However, this therapy is not successful for all patients and the mechanisms underlying anti-PD1 blockade need to be explored. Important breakthroughs are continuously being made such as the identification of mutations in *JAK1*, *JAK2* and β2-microglobulin genes associated to primary and acquired resistance to PD1 blockade therapy [2, 3].

PD1 is a type I transmembrane protein preferentially expressed in immune cells such as T, B and NK cells. PD-L1 is a member of the B7 family of co-stimulatory/co-inhibitory molecules of antigen presentation expressed by a wide range of cell types, including cancer cells. When engaged to its receptor PD-L1, PD1 strongly interferes with T cell receptor (TCR) signal transduction through several poorly understood molecular mechanisms. PD1 is made of an extracellular immunoglobulin-like binding domain, a transmembrane region and a cytoplasmic domain containing an immunoreceptor tyrosine-based inhibitory motif (ITIM) and an immunoreceptor tyrosine-based switch motif (ITSM) [4]. These motifs are implicated in its immunosuppressive effects. Interfering with PD1 signal transduction either by antibody blockade or any other means enhances T cell functions by potentiating signal transduction from the TCR signalosome. Here we review the known molecular pathways by which PD1 exerts its immunosuppressive functions in T cells.

## ANTIGEN PRESENTATION TO THE T CELL

**Figure 1:**
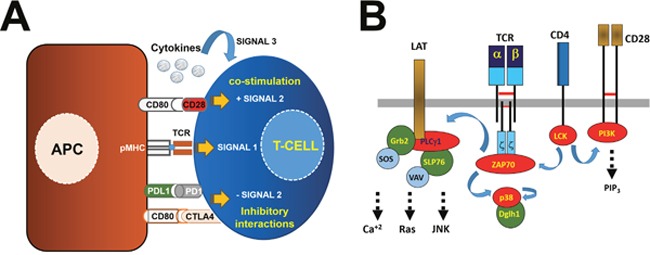
Antigen presentation and T cell activation through the T cell receptor signalosome **(A)** T cells receive from antigen presenting cells (APCs, left of the picture) three signals. APCs present antigenic peptides complexed to MHC molecules (pMHC, as indicated) to T cells through binding to their TCR as shown. This interaction triggers intracellular signaling events (signal 1) as indicated within the T cell on the right. T cells simultaneously receive additional “positive” and “negative” signals through ligand-receptor interactions within the immunological synapse. On top is shown the co-stimulatory interaction driven by CD80-CD28, and below two inhibitory interactions between PD-L1/PD1 and CD80-CTLA4. The integration of these signals delivers a second signal regulating the extent of T cell activation. A third signal is also provided by cytokines (top). **(B)** Simplified scheme of the TCR signalosome including the αβ TCR chains associated to CD3 molecules as indicated. In this example, only CD3ζ dimers are shown. Signal 1 is initiated through αβ TCR and CD4/CD8 molecules. This signal depends on LCK activation that phosphorylates CD3 and CD28 cytoplasmic domains. ZAP70 then binds to CD3ζ and phosphorylates LAT and p38 as shown. Phosphorylated LAT recruits other enzymes and adaptor molecules as shown which will trigger calcium-dependent and MAPK-dependent pathways. Signal 2 depends on PI3K associated to CD28 when associated to CD80 on the surface of the APC. PI3K generates PIP_3_ leading to proliferation and survival by regulating AKT-mTOR pathways. CD28 engagement prevents apoptosis and acts synergistically with CD3-dependent signals. In green, adaptor molecules. In red, kinases and phospholipase C.

T cell activation and expansion is a complex process regulated by the interaction of several signaling pathways. T cells get activated and expand exponentially after encountering antigenic peptides specific for their cognate TCRs. Antigenic peptides are presented to T cells usually by professional antigen presenting cells (APCs) such as dendritic cells (DCs) through a highly regulated process called antigen presentation (Figure 1A). APCs capture and intracellularly process antigens from pathogens and cancer cells into short antigenic peptides which are loaded into major histocompatibility molecules (peptide-MHC complexes, or pMHC) and then exposed to the surface. There, peptide-MHC complexes bind to the TCR within the immunological synapse (Figure 1A). This recognition entails the initial step of the activation of the TCR signaling cascade (signal 1). However, T cells need at least a second co-stimulatory signal to escape from anergy or apoptosis [5]. A wide range of stimulatory or inhibitory interactions between receptors on the T cell surface and their ligands on the surface of the APC lead to signal 2. Thus this signal 2 will determine the level of T cell activation. Possibly the most important co-stimulatory interaction is provided by CD80 on the APC binding to CD28 on the T cell. There is also a third signal delivered by cytokines which regulates T cell differentiation and effector capacities [6]. Then, other inhibitory interactions take place between the APC and the T cell which modulate the strength and duration of the stimulatory signals.

## MECHANISMS OF T CELL ACTIVATION

At the molecular level, signal one is delivered by kinase-dependent pathways triggered when TCR-CD3 molecules are bound to pMHC complexes in the immunological synapse (Figure 1A). TCR-CD3 and co-receptor (CD4 or CD8) cross-linking results in tyrosine phosphorylation of the TCR-CD3 intracellular domains by LCK and FYN kinases (Figure 1B). LCK also phosphorylates ZAP70 kinase which is then recruited to the CD3ζ chain [7]. ZAP70 starts multiple signaling events through LAT phosphorylation and association to GRB2 and PLCγ1, culminating with activation of the MAPKs ERK and JNK [8] involved in several cellular processes such as proliferation, differentiation, motility, stress response, apoptosis, and survival. PLCγ1 produces diacyl glycerol (DAG) and inositol 1,4,5-triphosphate (IP_3_) causing release of calcium ions from the ER and inducing NFAT and CREB translocation, which enhances IL2 transcription. Additionally, ZAP70 phosphorylates p38 associated to the scaffold protein DLGH1, resulting in its autophosphorylation at the activation loop by the so-called “alternative p38 activation pathway” which contributes to proliferation and cytokine production [9, 10].

Signal 2 (T cell co-stimulation) is exemplified by CD80-CD28 interactions between APCs and T cells (Figure 1). In this situation, LCK phosphorylates CD28 intracellular domain, providing a docking site for the PI3K complex. PI3K then generates phosphatidylinositol 3,4,5-triphosphate (PIP_3_), that activates downstream kinases including AKT which enhances proliferation and survival through the mTOR pathway [11], and PKCθ which activates NF-κB and MKK7, required for IL2 production. T cells require AKT and PKCθ activation to acquire full proliferative and effector activities.

Nevertheless, there are other associations between ligands and receptors in APCs and T cells (Figure 1A). Many of these will inhibit T cell activation to modulate their activities and have been extensively reviewed elsewhere [12].

## DIRECT PATHWAYS OF PD1-DEPENDENT INHIBITION OF TCR SIGNAL TRANSDUCTION

**Figure 2:**
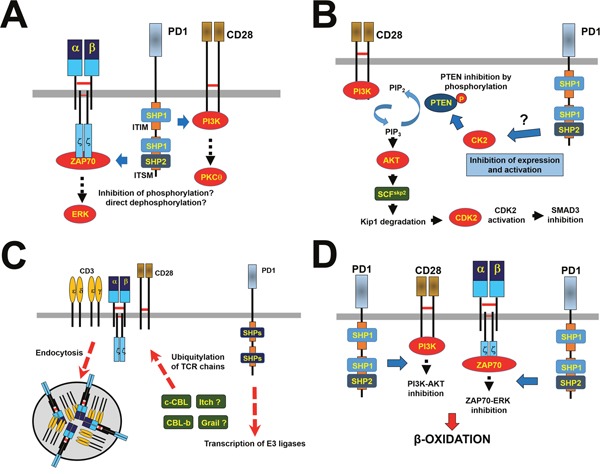
PD1-dependent inhibitory mechanisms **(A)** Direct inhibitory mechanisms over the TCR signalosome are shown. The figure represents PD1-dependent proximal inhibitory mechanisms, which depend on the recruitment of SHP1 and SHP2 phosphatases as shown. These phosphatases inhibit ZAP70 and PI3K activities (blue arrows). Downstream intracellular pathways are also terminated, as exemplified in the figure with ERK and PKCθ. **(B)** Indirect inhibitory mechanisms over TCR signaling and T cell proliferation are shown through regulation of CK2 expression and activities. On the left, the PI3K-dependent signaling pathway activating CDK2 and inhibiting SMAD3 is shown. Briefly, PIP3 activates AKT leading to production of the ubiquitin ligase SCF that degrades the CDK2 inhibitor Kip1. Activated CDK2 triggers cell cycle progression and inactivates SMAD3 by phosphorylation. These pathways are negatively regulated by the PTEN phosphatase that degrades PIP_3_. During TCR activation CK2 phosphorylates PTEN with a concomitant decrease in its activities. When PD1 is engaged CK2 expression and activities decrease resulting in active PTEN that eliminates PIP_3_ shutting off AKT activation. **(C)** Regulation of TCR surface expression by PD1. PD1 engagement promotes expression of E3 ubiquitin ligases of the CBL family, as shown. As indicated, other ligases may be up-regulated as well. These ubiquitin ligases ubiquitylate TCR chains and PI3K, leading to the removal of TCRs from the T cell surface, possibly by endocytosis. Thus, T cells cannot respond to antigenic stimulation. **(D)** Metabolic control by PD1. Engaged PD1 alters T cell metabolism from glycolysis to β-oxidation by inhibition of ERK and PI3K-AKT activities. PD1-stimulated T cells would then metabolically resemble long-lived memory T cells.

Similarly to CD80-CTLA4, PD-L1/PD1 interactions are antagonists of CD80-CD28 co-stimulation. PD-L1-engaged PD1 strongly counteracts TCR signal transduction and CD28-co-stimulation even at very low PD1 expression levels. As a result, PD1 abrogates cytokine production, causes cell cycle arrest and decreases transcription of the pro-survival factor Bcl-X_L_ [13]. In the direct pathway of TCR inhibition, engaged PD1 terminates ZAP70 and PI3K phosphorylation by recruiting SHP1 and SHP2 phosphatases to its tyrosine phosphorylated ITIM and ITSM motifs [14, 15] (Figure 2A). However, the specific mode of action is still poorly understood. Interestingly, only inactive mutations in the phosphorylatable ITSM tyrosine residues abolish PD1-inhibitory effects, but not in the ITIM motif [13]. SHP1 can also bind both ITIM and ITSM motifs, while SHP2 only binds the ITSM [14]. Nevertheless, whether SHP1 and SHP2 can simultaneously bind the ITSM is yet unknown (Figure 2A). It might be possible that binding of one prevents binding of the other, behaving like a regulatory switch motif similar to the ITSM of CD150 [16] that may regulate PD1 activities. In agreement with this, SHP2 but not SHP1 has been observed associated to PD1 in microclusters within the immunological synapse [17]. All these evidences support SHP2 as the main driver of PD1 inhibitory functions. Unlike SHP2, it is yet unclear whether PD1-bound SHP1 really contributes to PD1 suppressive function [17]. Nevertheless, SHP1 can bind to tyrosine-phosphorylated substrates such as ZAP70 [18]. It is thought that it could follow a pathway similar to termination of “standard” TCR signal transduction upon T cell activation through SHP1 dephosphorylating ZAP70 [18]. Even so, it has to be remarked that direct dephosphorylation of TCR proximal signaling kinases by SHP1 and SHP2 associated to PD1 has not yet been demonstrated [14].

Engaged PD1 has to be recruited to the immunological synapse to exert its activities. Thus, TCR engagement with pMHC is sufficient to cause SHP2 recruitment to PD1, but no T cell suppressive effects are exerted unless PD1 is simultaneously ligated [13]. This would suggest that PD1-associated phosphatases need the proximity to the TCR signalosome to dephosphorylate signal transduction kinases in analogy to CTLA4 recruiting SHP2 to dephosphorylate CD3ζ [13, 19]. The consequent inhibition of TCR-dependent distal signaling pathways such as ERK are probably the result of direct ZAP70 inhibition (Figure 2A).

## INDIRECT PATHWAYS OF PD1-DEPENDENT INHIBITION OF TCR SIGNAL TRANSDUCTION

PD-L1-engaged PD1 exerts an additional indirect inhibitory control over CD28-costimulation by regulating the expression levels and activities of CK2 and cyclin-dependent kinases (CDKs) (Figure 2B) [20]. In the absence of PD1 engagement, TCR activation increases CK2 expression that inactivates PTEN by phosphorylation. Thus, PTEN cannot dephosphorylate PIP3 produced by PI3K allowing TCR signal transduction to proceed. Interestingly, engaged PD1 reduces CK2 expression and activity with the result of an active PTEN that terminates the PI3K-AKT- PKCθ pathway inhibiting T cell growth and survival [14].

Engaged PD1 arrests lymphocytes at the G0-G1 phase through an indirect pathway which inhibits CDKs (Figure 2B). The resulting inhibition of AKT and ERK either by direct or indirect pathways inhibits transcription of SKP2 (part of SCF^skp2^ ubiquitin ligase) (Figure 1A and 1B) [21]. SCF^skp2^ ubiquitin ligase normally tags the CDK2 inhibitor p27^Kip1^ to proteosomal degradation during T cell activation, driving CDK2-dependent cell division. Thus, PD1 activities cause an accumulation of p27^Kip1^ that associates with CDK2 inhibiting its kinase activities. CDK2 inhibition also eliminates the SMAD3 inhibiting phosphorylation on Ser213, resulting in the expression of SMAD3-responsive genes including p15^INK4B^ (a potent inhibitor of CDK4 and CDK6), and repressing transcription of the tyrosine phosphatase CDC25A that normally removes inhibitory tyrosine phosphorylations of CDK4, CDK6 and CDK2 causing T cell arrest [22].

### PD1 regulation of TCR down-modulation

Down-modulation and degradation of the TCR signalosome is a feature of T cells in cancer patients and animal cancer models, especially in tumor-infiltrating lymphocytes [23–25]. The most effective way to prevent T cells from recognizing antigens is to remove TCRs from their surface. Then, T cell function is severely impaired as a direct consequence of down-modulated TCRs and their associated receptors and signaling mediators (signalosome, Figure 1B and 2C). Cancer-associated TCR down-modulation is not caused by low affinity interactions with tumor antigens. This was demonstrated in a C-MYC-dependent OVA-expressing hepatocarcinoma murine model. Prolonged TCR down-modulation was observed in adoptively transferred OVA-specific transgenic CD8 OTI cells which have high affinity TCRs [26]. Thus, other mechanisms must be in place.

PD1 expression is characteristically high in tumor-infiltrating T cells [27, 28]. Thus, PD-L1-engaged PD1 contributes to prolonged TCR down-modulation by up-regulating the expression of E3-ubiquitin ligases CBL-B, c-CBL and ITCH which ultimately causes TCR internalization [29, 30] (Figure 2C). Interfering with PD-L1/PD1 interactions either with blocking antibodies or by gene silencing during antigen presentation to T cells leads to hyperactivated TCR^high^ T cells with decreased expression of c-CBL and CBL-b [30].

All the evidence shows that E3 ubiquitin ligases are closely linked to regulation of TCR levels and termination of TCR-dependent signal transduction. CBL-b and c-CBL double-knockout T cells exhibit impaired TCR down-modulation upon T cell activation, causing hyperactivation in the absence of CD28 co-stimulation [31]. CBL-b single knock-out T cells also show impaired TCR down-modulation during physiological immune responses or after antigen recognition, similarly to disruption of PD-L1/PD1 interactions [30, 32]. Mechanistically, CBL-b and ITCH cause K33-polyubiquitination of CD3ζ, impairing its phosphorylation and the subsequent association of ZAP70 [33]. PI3K is also a direct target for CBL-b causing proteolysis-independent PI3K inactivation [34]. Overall, CBL-b negatively regulates CD28 co-stimulation in T cells [35, 36]. Thus, it is highly likely that the strong CD28-counteracting activities of PD1 could be partly caused by E3 ubiquitin ligases. It is worth noting that PD-L1 blockade also inhibits down-modulation of additional T cell activation markers such as ICOS, making PD1-driven receptor down-modulation a major regulatory mechanism of T cell functions [30, 37].

### PD1 control of T cell metabolism

It is well known that T cell activation requires a fast energy increase which is achieved by glycolysis-dependent metabolism. Interestingly, engaged PD1 may be fundamental to rapidly shift the metabolic reprogramming of T cells from an effector to a long-lived memory-like phenotype with a shift from increased glycolysis towards fatty acid β-oxidation [38, 39] (Figure 2D). It is thought that this metabolic change might extend the lifespan of PD1^high^ T cells. The exact molecular mechanisms are still unclear, although it could be mediated by PD1-dependent inhibition of PI3K and ERK. Thus, simultaneous inhibition of PI3K/AKT and ERK recapitulated some of PD1-dependent metabolic changes [39]. It is remarkable that PD1-engaged T cells closely resemble long-lived memory T cells from a metabolic point of view, when the current view is that sustained PD1 stimulation leads to T cell exhaustion. The implications of PD1 signaling in T cell differentiation will probably expand the accepted roles of PD1 as an immunosuppressive pathway.

## FUNCTIONAL CONSEQUENCES OF PD-L1/PD1 DISRUPTION

**Figure 3:**
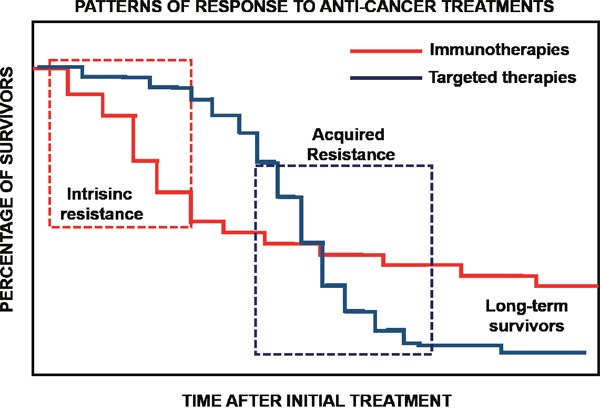
Immunotherapies and targeted therapies display distinct patterns of response The graph displays a model of responses for immunotherapies and targeted therapies such as kinase inhibitors. The percentage of survival is plotted over time after the initial treatment. While targeted therapies exhibit fast anti-tumor effects, tumors acquire resistance to treatments. This is exemplified as a sudden drop of survival as indicated within the dotted square on the right. In contrast, immunotherapies show a significant group of patients that are intrinsically resistant to therapy, evidenced as an initial sudden drop in survival as indicated within the dotted square on the left. Then, long-term survivors are found with durable responses. So far, there are not effective biomarkers discriminating patients that could benefit from immunotherapies from those with intrinsic resistance.

The immune response is highly complex, and many factors can influence the outcome of immunotherapies. In addition, response patterns of PD-L1/PD1 blockade therapy are strikingly different from those of chemotherapy or targeted therapies. Targeted therapies cause fast tumor remissions but with prompt acquisition of resistance (Figure 3). In contrast, patients who respond to PD-L1/PD1 blockade show significant and durable responses, although a delay of even months can be observed. From a clinical point of view there is a strong need to identify biomarkers that can predict the outcome of PD-L1/PD1 blockade. It is naturally assumed that PD-L1 expression in cancer cells would correlate with therapeutic outcome. However, the results from clinical trials differ on the usefulness of PD-L1 expression as a biomarker, as patients with PD-L1-positive and PD-L1-negative tumors may benefit from the treatment [1].

To explain the apparent contradictions observed in clinical trials we have first to consider that PD-L1/PD1 functions are context-specific. The regulatory functions of this interaction take place in at least three stages: (i) During antigen presentation to T cells [30]; (ii) in peripheral tissues to maintain tolerance [40, 41], and (iii) at the inflammation site to dampen excessive autoreactive damage [4, 40]. Thus, systemic disruption of PD-L1/PD1 interactions in cancer patients will unleash several events contributing to therapeutic efficacy but also to side effects:

### Hyperactivation of naïve T cells undergoing antigen presentation

Systemic PD-L1/PD1 blockade very likely affects naïve T cells undergoing antigen presentation by professional APCs in lymphoid organs, as PD-L1 is normally highly expressed by professional APCs such as DCs [30, 40, 42]. PD-L1 expression is strongly up-regulated following DC maturation probably as a regulatory negative feedback mechanism. However, PD-L1/PD1 interactions play a critical role in physiological T cell activation during antigen presentation. Engaged PD1 in T cells during antigen presentation by DCs causes prolonged ligand-induced TCR down-modulation, which is followed by exponential clonal expansion rather than permanent T cell inactivation [30]. TCR surface expression is recovered within two weeks when a significant number of antigen-specific T cells have accumulated, ready to exert cytotoxic activities over target cells [43]. Disruption of PD-L1/PD1 interactions at this stage leads to hyperactivated TCR^high^ effector T cells which mediate an early immune attack [43, 44]. The resulting expansion of activated T cells from the pool of naïve T cells may enhance the anti-tumor response. Some evidence in preclinical models has been recently published which suggests that this could in fact be the case [45]. Moreover, interference of PD-L1/PD1 interactions during antigen presentation by DCs also favors that T cells do not lose their capacities to simultaneously express more than one cytokine (such as TNF-α, IL2, IFN-γ and MIP-1β) [42], in agreement with the capacities of PD-L1/PD1 blockade to revert (or prevent) T cell exhaustion [46].

### Recovery of T cell functions of cancer-specific memory T cells

Tumors are infiltrated with an array of immune cells which includes memory PD1+ T cells. These T cells are antigen-experienced but they have been inactivated by the tumor [47]. Reactivation of these experienced memory T cells might be the cause of the efficacy of PD-L1/PD1 blockade in patients with PD-L1-positive tumors [48]. These intra-tumor T cells usually lack expression of the TCR signalosome, and PD-L1/PD1 blockade may contribute to the recovery of TCR surface expression and signal transduction. The reactivation of this pool of antigen-experienced T cells seems to be the driver of the efficacy of PD-L1/PD1 blockade in human therapy [49]. In fact, T cell infiltration can be used as a predicting factor. Tumor infiltration with PD1^high^ CTLA4^high^ exhausted CD8 T cells can be an accurate predictor of response to anti-PD1 therapy in melanoma [50, 51].

### Polyclonal expansion of T cells in peripheral tissue

PD-L1/PD1 interactions in peripheral tissues between many cell types and T cells ensure that circulating T cells do not react with autoantigens. This is also reinforced by the decreased expression of surface TCR in activated T cells during the clonal expansion phase [30, 43]. However, systemic disruption of PD-L1/PD1 may have a significant impact on antigen recognition in peripheral tissue. In fact, it has been shown that local PD-L1 silencing in peripheral tissue expands polyclonal CD8 T cells [41] suggesting that systemic administration of PD-L1/PD1 blocking antibodies may expand a polyclonal pool of T cells following antigen recognition in peripheral tissues. The nature of these T cells has not been elucidated yet, but these reactivated T cells may be behind of some of the autoreactive adverse events associated to treatments with immune checkpoint inhibitors [52, 53]. Nevertheless, these T cells may also contain a significant pool of tumor antigen-specific cells that can be recruited to the tumor environment.

### Direct effects over cancer cells

The current conventional view states that direct disruption of PD-L1/PD1 interactions between cancer and T cells is behind the therapeutic efficacy of blocking antibodies. However, PD-L1/PD1 interactions may also take place between melanoma cells in the absence of T cells [54]. This suggests that a minor population of melanoma cells may express some PD1 on their surface. Elimination of PD1 expression in murine mouse melanoma models and also in human xenografts inhibited cancer cell growth. Antibody-mediated disruption of this interaction directly delayed tumor growth without the need of enhanced T cell anti-tumor activities. The same result was achieved by either PD1 silencing or PD-L1 silencing in tumor cells [41, 54]. Thus, intercellular PD-L1/PD1 interactions transmit survival signals to cancer cells and promote *in vivo* tumor growth Therefore, disruption of PD-L1/PD1 interactions between cancer cells will delay tumor growth while anti-cancer T cells become activated [41].

## CONCLUSIONS

PD-L1/PD1 antibody blockade has yielded encouraging results for the treatment of a wide range of cancer types. The exact molecular and cellular mechanisms behind its potent anti-cancer activities are still poorly understood. PD1 engagement by PD-L1 leads to rapid termination of TCR intracellular signaling and inhibition of T cell proliferation through direct and indirect mechanisms. Most of the evidence points to SHP-2 recruitment as the main driver of all these mechanisms. However, it has to be mentioned that many experimental evidences so far come from immortal Jurkat T cells. Therefore, it cannot be ruled out that PD1 employs other yet unknown pathways to disrupt TCR signaling cascades and alter T cell metabolism.

A key issue with PD-L1/PD1 blockade therapy is the identification of predictive markers of response. Recently, inactivating mutations in the interferon signal transduction pathway and in class I antigen presentation have been shown to correlate with both primary and adaptive resistance to anti-PD1 therapy treatment [2, 3]. Therefore, there is a frantic need to identify alternative targets susceptible of therapeutic intervention that could minimize cancer cell escape. The tumor environment is a complex system in which many cell types cooperate to protect it, including myeloid-derived suppressor cells, tumor-infiltrating macrophages, regulatory T cells and other cell types of the tumor stroma [55–58]. Many of these cell types utilize the PD-L1/PD1 signaling axis to suppress anti-tumor immune responses. Therefore, the understanding of the mechanisms behind PD1-dependent cell suppression will surely uncover a number of novel targets susceptible of therapeutic intervention.

During the last years, therapies targeting main regulatory components of the immune synapse have been developed and are progressively displacing chemotherapy in different clinical contexts [1, 51, 59, 60]. Preliminary results from the simultaneous blockade of PD1 and CTLA4 in metastatic melanoma were also published, and now this combination is being tested in many different neoplasms [61, 62]. The better understanding of the signaling pathways underlying the immune response has revealed potential targets that might improve the efficacy of the current treatments. Many new drugs are now under evaluation, both potentiating co-stimulatory molecules such as 4-1BB, OX40, CD27, CD40, GITR, or blocking immunosuppressive proteins including LAG-3 or VISTA [63–65]. Their efficacy in combination with PDL1/PD1 blockade and CTLA4-targeted therapies will surely be reported in the near future.
